# Supplementary material for: Effect of non-pharmaceutical interventions in the early phase of the COVID-19 epidemic in Saudi Arabia
Source: PLOS Glob Public Health. 2022 May 9;2(5):e0000237. doi: 10.1371/journal.pgph.0000237 (PMC10021433; doi:10.1371/journal.pgph.0000237)
Supplement: S1 Table — Description of fixed and inferred parameters. (DOCX) [file pgph.0000237.s001.docx]

| **Table S1 Model parameter values** | |  |
| --- | --- | --- |
| **Fixed Parameters** | **Range or value** | **Reference** |
| Incubation time | 5.2 days | [[1]](https://paperpile.com/c/7MJin7/9dhG) |
| Serial interval | 7.2 days | [[2,3]](https://paperpile.com/c/7MJin7/vLrs+oTqR) |
| Infection fatality risk | 0.3-0.5% depending on regions after age-standardization according to the region’s age distribution | [[24]](https://paperpile.com/c/7MJin7/NsaR) |
| Delay between infection and death | 25 days | [[5]](https://paperpile.com/c/7MJin7/N1q5) |
| Delay between infection and case confirmation | 3 days | [[5]](https://paperpile.com/c/7MJin7/N1q5) |
| **Inferred Parameters**  (region-dependent) |  |  |
| Probability of case reporting given infection | 8% - 50% (Table S3) |  |
| Infection seeding date and amount in each region | Seeding date February 18 - April 6 Seeding amount 14 - 100 (Table S4) |  |
| Effect of NPIs in reduction of the effective reproductive number | Table S2 |  |
