# Supplementary material for: Effect of non-pharmaceutical interventions in the early phase of the COVID-19 epidemic in Saudi Arabia
Source: PLOS Glob Public Health. 2022 May 9;2(5):e0000237. doi: 10.1371/journal.pgph.0000237 (PMC10021433; doi:10.1371/journal.pgph.0000237)
Supplement: S2 Table — Estimates are given in terms of the mean and the 95% CrI. (DOCX) [file pgph.0000237.s002.docx]

| **Table S2: NPI efficiency estimates. Estimates are given in terms of the mean and the 95% CrI.** | | | | | | | | |
| --- | --- | --- | --- | --- | --- | --- | --- | --- |
| **Region** | **Isolation and**  **School Closure** | **Curfew**  **(first half)** | **Curfew**  **(second half)** | **Lockdown** | **Phase I** | **Phase II** | **Phase III**  **(first half)** | **Phase III**  **(second half)** |
| Al Bahah | 0.4  (0.034-0.73) | 0.51 (0.11-0.85) | 0.4 (0.024-0.8) | 0.58  (0.054-0.93) | 0.35 (0.014-0.85) | 0.25 (0.011-0.66) | 0.51 (0.17-0.79) | 0.48 (0.067-0.82) |
| Al Hudud  Ash Shamaliyah | 0.38  (0.021-0.73) | 0.37 (0.028-0.81) | 0.46 (0.044-0.83) | 0.6  (0.087-0.94) | 0.36 (0.016-0.85) | 0.31 (0.016-0.73) | 0.48 (0.16-0.79) | 0.54  (0.16-0.83) |
| Al Jawf | 0.35  (0.015-0.73) | 0.48 (0.023-0.89) | 0.6 (0.061-0.89) | 0.59  (0.066-0.94) | 0.4 (0.023-0.88) | 0.28 (0.013-0.82) | 0.57 (0.15-0.88) | 0.63 (0.082-0.89) |
| Al Madinah | 0.38  (0.022-0.73) | 0.29 (0.018-0.69) | 0.65 (0.13-0.89) | 0.59  (0.054-0.94) | 0.41 (0.027-0.86) | 0.45 (0.082-0.83) | 0.62 (0.32-0.86) | 0.49  0.1-0.82) |
| Al Quassim | 0.36  (0.021-0.74) | 0.35 (0.025-0.67) | 0.42 (0.027-0.84) | 0.59  (0.086-0.93) | 0.38 (0.024-0.86) | 0.23 (0.0073-0.61) | 0.63 (0.38-0.85) | 0.65  (0.21-0.88) |
| Ar Riyad | 0.3  (0.012-0.69) | 0.24 (0.011-0.63) | 0.33 (0.019-0.74) | 0.58  (0.062-0.93) | 0.38 (0.016-0.86) | 0.49 (0.049-0.85) | 0.67 (0.46-0.86) | 0.58  (0.12-0.86) |
| Ash Sharqiyah | 0.36 (0.024-0.72) | 0.24 (0.01-0.65) | 0.41 (0.03-0.81) | 0.59  (0.075-0.93) | 0.37 (0.014-0.85) | 0.34 (0.018-0.74) | 0.61 (0.38-0.82) | 0.57  (0.21-0.85) |
| Asir | 0.41  (0.019-0.74) | 0.55 (0.098-0.85) | 0.34 (0.022-0.79) | 0.53  (0.032-0.93) | 0.36 (0.014-0.87) | 0.26 (0.0098-0.66) | 0.58 (0.22-0.83) | 0.62  (0.19-0.88) |
| Hail | 0.35  (0.011-0.72) | 0.47 (0.022-0.86) | 0.39 (0.041-0.8) | 0.6  (0.046-0.93) | 0.36 (0.026-0.85) | 0.21 (0.011-0.61) | 0.63 (0.39-0.84) | 0.62  0.18-0.87) |
| Jizan | 0.41  (0.013-0.74) | 0.35 (0.036-0.75) | 0.42 (0.036-0.81) | 0.56 (0.073-0.92) | 0.38 (0.018-0.85) | 0.28 (0.013-0.69) | 0.55 (0.26-0.79) | 0.69  (0.24-0.89) |
| Makkah | 0.25  (0.011-0.69) | 0.23 (0.013-0.59) | 0.51 (0.06-0.84) | 0.59 (0.081-0.94) | 0.55 (0.16-0.87) | 0.51 (0.071-0.82) | 0.6  (0.34-0.83) | 0.58  (0.12-0.88) |
| Najran | 0.4  (0.03-0.74) | 0.7  (0.11-0.9) | 0.3  (0.01-0.84) | 0.49  (0.026-0.92) | 0.4 (0.012-0.86) | 0.29 (0.016-0.77) | 0.59  (0.1-0.86) | 0.55 (0.094-0.88) |
| Tabuk | 0.37  (0.02-0.73) | 0.32 (0.035-0.73) | 0.45 (0.031-0.86) | 0.6  (0.066-0.93) | 0.39 (0.023-0.86) | 0.27  0.01-0.67) | 0.58 (0.32-0.82) | 0.68  (0.16-0.89) |
