# Supplementary material for: Effect of non-pharmaceutical interventions in the early phase of the COVID-19 epidemic in Saudi Arabia
Source: PLOS Glob Public Health. 2022 May 9;2(5):e0000237. doi: 10.1371/journal.pgph.0000237 (PMC10021433; doi:10.1371/journal.pgph.0000237)
Supplement: S3 Table — Estimates given in terms of the mean and the 95% CrI. (DOCX) [file pgph.0000237.s003.docx]

| **Table S3: Region-specific estimated confirmation probabilities.** Estimates given in terms of the mean and the 95% CrI. | |
| --- | --- |
| **Region** | **Estimated confirmation rate** |
| Al Bahah | 0.09 (0.011-0.25) |
| Al Hudud   Ash Shamaliyah | 0.084 (0.0097-0.25) |
| Al Jawf | 0.53 (0.071-0.97) |
| Al Madinah | 0.47 (0.2-0.81) |
| Al Quassim | 0.24 (0.058-0.48) |
| Ar Riyad | 0.27 (0.065-0.66) |
| Ash Sharqiyah | 0.49 (0.22-0.84) |
| Asir | 0.31 (0.079-0.68) |
| Hail | 0.27 (0.063-0.6) |
| Jizan | 0.13 (0.019-0.31) |
| Makkah | 0.18 (0.045-0.38) |
| Najran | 0.26 (0.02-0.85) |
| Tabuk | 0.25 (0.048-0.55) |
