# Supplementary material for: Effect of non-pharmaceutical interventions in the early phase of the COVID-19 epidemic in Saudi Arabia
Source: PLOS Glob Public Health. 2022 May 9;2(5):e0000237. doi: 10.1371/journal.pgph.0000237 (PMC10021433; doi:10.1371/journal.pgph.0000237)
Supplement: S4 Table — Estimates given in terms of the mean and the 95% CrI. (DOCX) [file pgph.0000237.s004.docx]

| **Table S5: Region-specific estimated seeding dates and amounts.** Estimates given in terms of the mean and the 95% CrI. | | |
| --- | --- | --- |
| **Region** | **Seeding aount** | **Seeding date** |
| Al Bahah | 32 (3-124) | 2020-04-04 (2020-03-09-2020-05-03) |
| Al Hudud Ash Shamaliyah | 36 (5-84) | 2020-04-08 (2020-03-13-2020-05-02) |
| Al Jawf | 30 (4-85) | 2020-04-22 (2020-04-06-2020-05-09) |
| Al Madinah | 60 (3-195) | 2020-03-21 (2020-03-12-2020-04-04) |
| Al Quassim | 14 (3-26) | 2020-03-24 (2020-03-08-2020-04-09) |
| Ar Riyad | 44 (5-125) | 2020-03-09 (2020-02-25-2020-03-27) |
| Ash Sharqiyah | 22 (4-44) | 2020-03-02 (2020-02-18-2020-03-16) |
| Asir | 44 (2-145) | 2020-03-22 (2020-03-09-2020-04-13) |
| Hail | 18 (3-44) | 2020-04-16 (2020-03-31-2020-05-07) |
| Jizan | 20 (5-34) | 2020-03-24 (2020-03-04-2020-04-14) |
| Makkah | 102 (2-275) | 2020-03-07 (2020-02-23-2020-03-19) |
| Najran | 46 (4-85) | 2020-04-01 (2020-03-14-2020-05-15) |
| Tabuk | 16 (3-35) | 2020-03-23 (2020-03-05-2020-04-11) |
