# Supplementary material for: Effect of non-pharmaceutical interventions in the early phase of the COVID-19 epidemic in Saudi Arabia
Source: PLOS Glob Public Health. 2022 May 9;2(5):e0000237. doi: 10.1371/journal.pgph.0000237 (PMC10021433; doi:10.1371/journal.pgph.0000237)
Supplement: S5 Table — IFRs were calculated based on demographic characteristics of each region using the COVIDSeverity R package. (DOCX) [file pgph.0000237.s005.docx]

| **Table S5: Region-specific infection fatality ratios.** IFRs were calculated based on demographic characteristics of each region using the COVIDSeverity R package. | |
| --- | --- |
| **Region** | **IFR [%]** |
| Al Bahah | 0.57 |
| Al Hudud   Ash Shamaliyah | 0.39 |
| Al Jawf | 0.37 |
| Al Madinah | 0.46 |
| Al Quassim | 0.41 |
| Ar Riyad | 0.36 |
| Ash Sharqiyah | 0.35 |
| Asir | 0.51 |
| Hail | 0.47 |
| Jizan | 0.49 |
| Makkah | 0.45 |
| Najran | 0.4 |
| Tabuk | 0.36 |
