# Supplementary material for: Effect of non-pharmaceutical interventions in the early phase of the COVID-19 epidemic in Saudi Arabia
Source: PLOS Glob Public Health. 2022 May 9;2(5):e0000237. doi: 10.1371/journal.pgph.0000237 (PMC10021433; doi:10.1371/journal.pgph.0000237)
Supplement: S1 Text — Description of the MCMC algorithm used to infer model parameters. (DOCX) [file pgph.0000237.s006.docx]

# **Supplementary Text - Effect of non-pharmaceutical interventions in the early phase of the COVID-19 epidemic in Saudi Arabia**

Javier Perez-Saez, Elizabeth Lee, Nikolas Wada, Ada Alqunaibet^,^ Sami Almudarra, Reem Alsukait, Di Dong, Yi Zhang^,^ Sameh El Saharty, Christopher H. Herbst, Justin Lessler

# Inference

We built an inference framework to be able to calibrate simulated epidemic trajectories to observed epidemic data. Calibrated parameters of our modeling framework, which are all potentially location-specific, include epidemic seeding dates and amounts, values of the basic reproduction number, and the effectiveness of different types of NPIs. Inference on model parameters for each spatial location are drawn jointly given the spatial coupling of COVID-19 transmission dynamics.

Fit parameters are user specified, and can include NPI effectiveness and the timing and number of introduced cases that seed the epidemic.

## Pseudo-Likelihood

We enable the user to specify the likelihood function in terms of the epidemiological data used for calibration (e.g., number of incident confirmed cases or deaths) and the probability distributions associated with those calibration data. We consider the likelihood of the set of parameters for a location
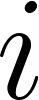
, 
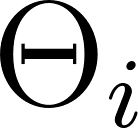
, given some set of
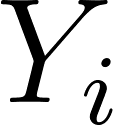
 to be equal to likelihood of that simulation given the data:


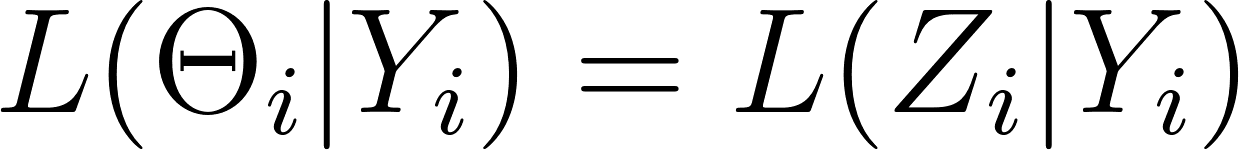


Where
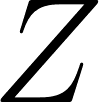
 is a full epidemic simulation (jointly generated for all locations) generated from a parameter set
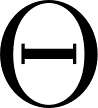
. Note that since epidemic simulations are not stochastic, this value will not be deterministically determined for any parameter set.

Given a location
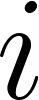
, a set of
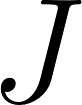
 data variables*,* and
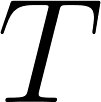
 time units (e.g., days, weeks), the likelihood of a simulated epidemic for a location
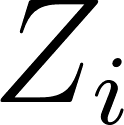
 given the observed data
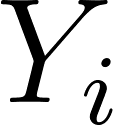
 at that location is :


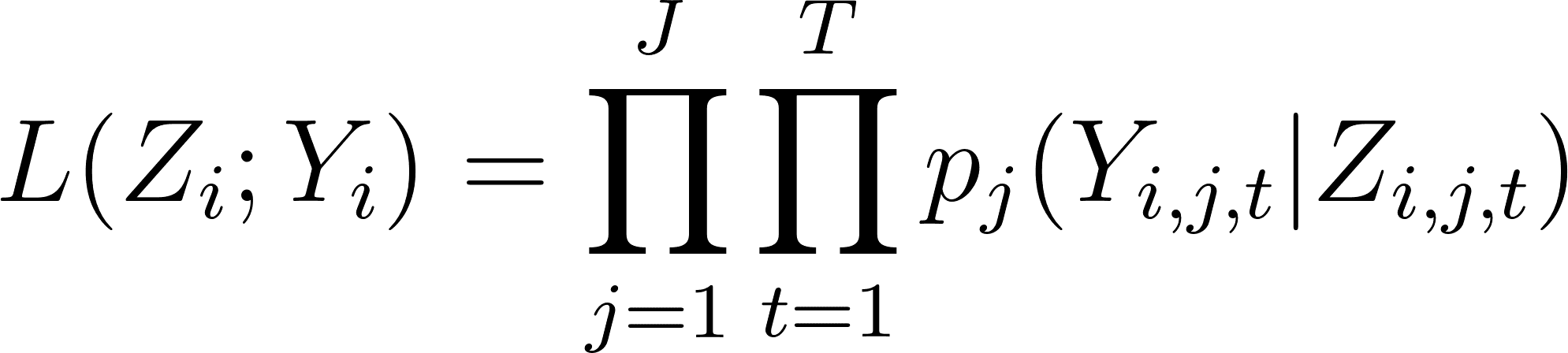


where
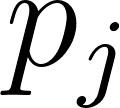
 is the user-specified probability distribution for data variable
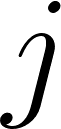
. These location-specific likelihoods to compute the full simulation likelihood are:


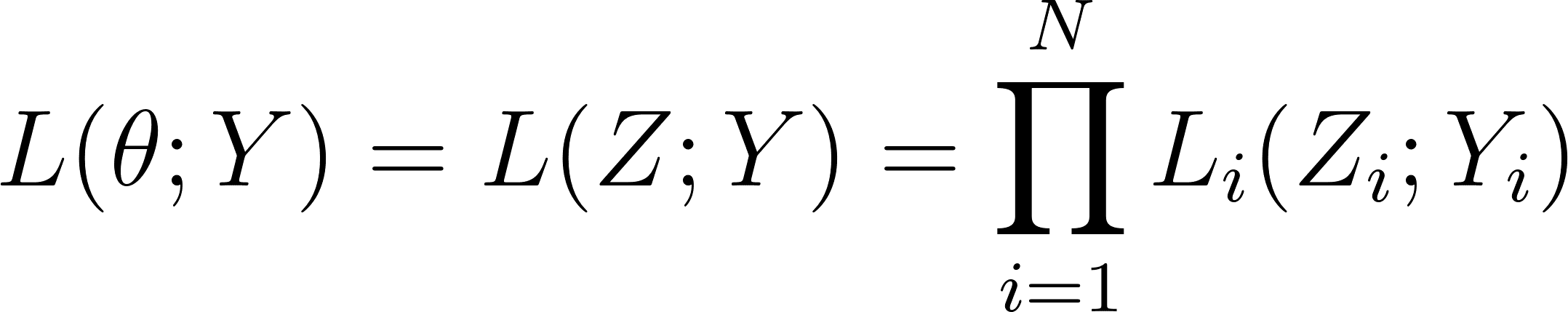


where
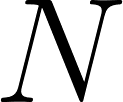
 is  the number of modeled locations. The framework allows the user to specify the time unit for a given calibration data; for instance one may calibrate to weekly incident deaths, biweekly confirmed cases, or both.

## Fitting Algorithm

We attempt to obtain a posterior distribution over both parameters
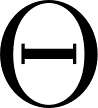
 and epidemic trajectories
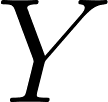
 using a modified block MCMC algorithm designed to take advantage of highly parallel computing resources. In its essence, this algorithm runs a larger number of relatively short MCMC chains, and uses the final accepted value from each chain to approximate the posterior distribution.

Each MCMC chain explores both parameter space and the space of epidemic trajectories in a way designed to efficiently explore parameter space in a way that aggressively maximizes individual local likelihoods, while preserving globally consistent epidemic trajectories. Each MCMC iteration
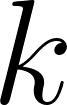
  proceeds as follows:

1. generate proposed parameter set
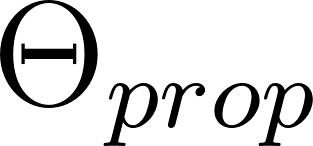
.
2. stochastically generate epidemic trajectory
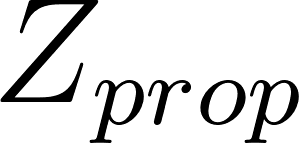
 using parameter set
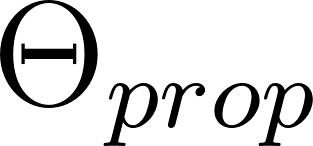

3. **if** (
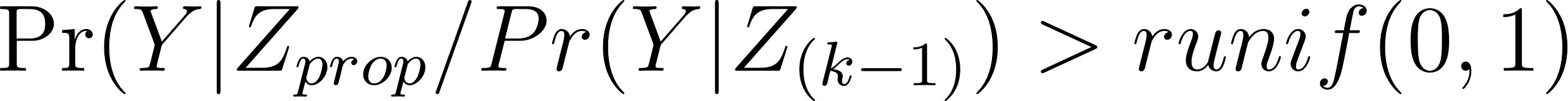
)

**set**
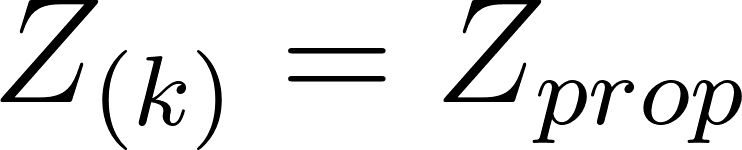


**set**
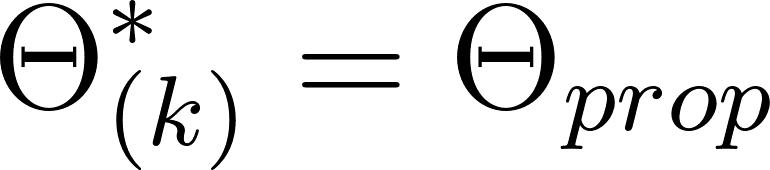


**else**

**set**
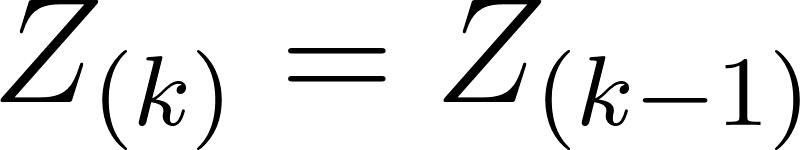


**set**
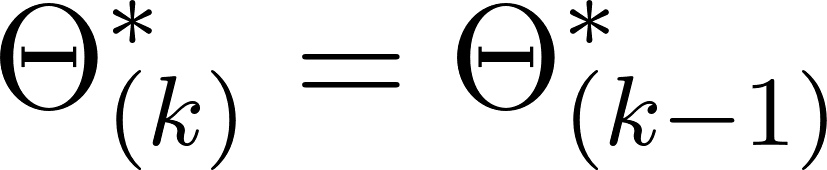


**endif**

1. **for** (i **in** 1...N)

**if** (
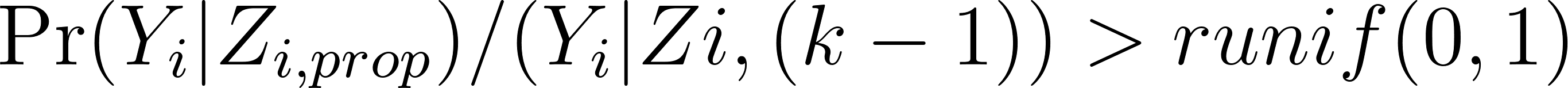
)

**set**
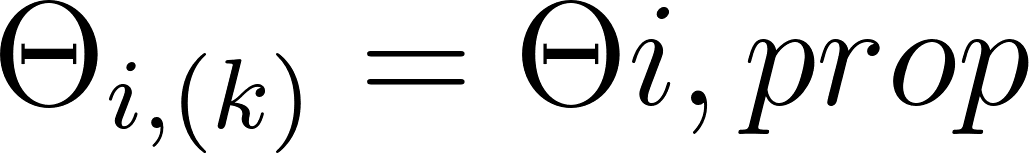


**else**

**set**
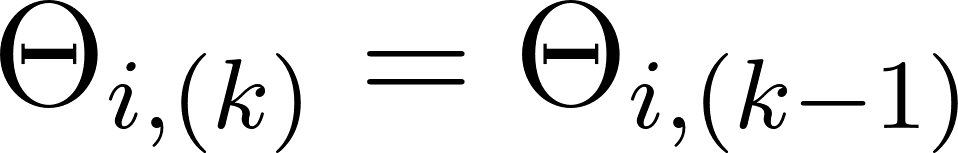


**endif**

**endfor**

This process is repeated a fixed number
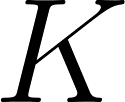
 times and for each of
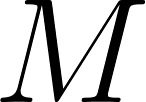
 slots, with
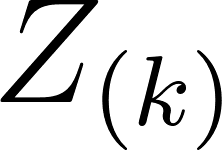
 and
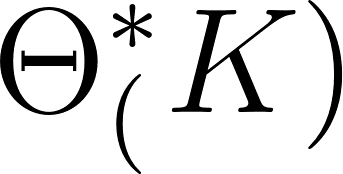
 being the final returned value for each slot. This final set of values is considered to represent both the posterior fit distribution of parameters and the posterior predictive distribution over epidemic trajectories. Proposal distribution (i.e., perturbations) for parameters can be specified in the config file. In this analysis we used 100 chains run in parallel for 300 iterations.

# Implementation

## Likelihood function

We use the floowing likelihood functions on the weekly cumulative number of death and cases:

$observedcases\sim Poisson\left( modeledcases \right)$

$\sqrt{\left( observeddeaths \right)}\sim Normal\left( \sqrt{\left( modeleddeaths \right)},\sqrt{\left( observeddeaths \right)}*0.075 \right).$

## Chain initialization

Chains were initialized using random starting conditions for each parameter as follows:

R0 ~ truncated-normal(mean = 2, sd = .1, limits = [1, 3])

Isolate and test ~ truncated-normal(mean = .15, sd = .1, limits = [0, .75])

Curfew ~ truncated-normal(mean = .2, sd = .1, limits = [0, .9])

Lockdown ~ truncated-normal(mean = .7, sd = .1, limits = [0, .95])

Phase I ~ truncated-normal(mean = .3, sd = .1, limits = [0, .9])

Phase II ~ truncated-normal(mean = .15 sd = .1, limits = [0, .9])

Phase III ~ truncated-normal(mean = .6, sd = .1, limits = [0, .9])

case confirmation rate ~ truncated-normal(mean = .3, sd = .1, limits = [0, 1])

## Priors

We set weakly informative normal(2.5, 1) priors on the basic reproductive numbers, and flat priors on all other model parameters.

## Comparison with seroprevalence estimates

We compare our modeling results to a seroprevalence study done in 2020 in Saudi Arabia [6]. We show in Figure S1 the comparison between the seroprevalence estimates in the six sampled regions and their corresponding fraction of cumulative infections implied by our mode. The serosamples were collected from Jun-Nov 2020, depending on the region. Riyadh was sampled in Jun 2020. Modeled cumulative infection rates tended to underestimate serology-based estimates for both blood donor and overall samples, in particular for serosurveys done in the second half of the simulation period. We note that our simulation period ends in October 2020, whereas serosurveys in Ash Sharqiyah and Makkah were done in November 2020 which may explain at least part of the underestimation. There is however a significant linear relationship between modeled infections and seroprevalence estimates, with approximately twice as many seroprevalence-based infections for each modeled infection. The systematic under-estimation could be caused by by an overestimation of the realized infection fatality ratio with respect to the estimates based on the region’s demographics, or the seroprevalence estimates could be over-estimated since the survey was based on a convenience sample.

| 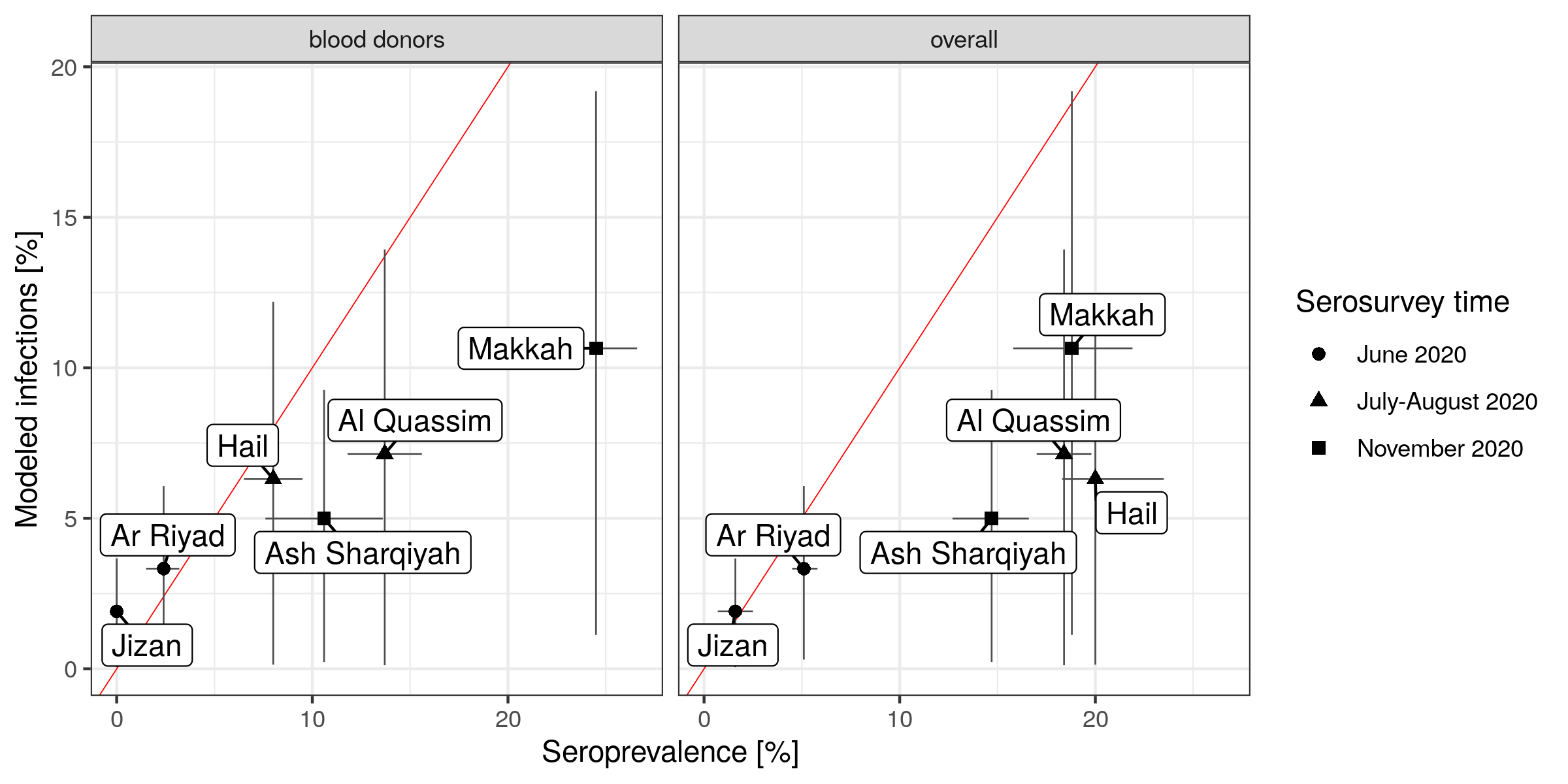 |
| --- |
| **Figure S1: Comparison between seroprevalence studies and modeled infections.** Seroprevalence data was extracted from [6], with estimates stratified by sampled population (blood donors and blood donors + non-COVId-19 patients). Cumulative modeled infections were matched to the midpoint of the serosurvey period when it fell within the simulation time window, and set to the end of the simulation period (Oct 31 2020) otherwise. Error bars indicate the 95% uncertainty intervals. |

# References

1. [Lauer SA, Grantz KH, Bi Q, Jones FK, Zheng Q, Meredith HR, et al. The Incubation Period of Coronavirus Disease 2019 (COVID-19) From Publicly Reported Confirmed Cases: Estimation and Application. Ann Intern Med. 2020;172: 577–582.](http://paperpile.com/b/7MJin7/9dhG)

2. [Ali ST, Wang L, Lau EHY, Xu X-K, Du Z, Wu Y, et al. Serial interval of SARS-CoV-2 was shortened over time by nonpharmaceutical interventions. Science. 2020;369: 1106–1109.](http://paperpile.com/b/7MJin7/vLrs)

3. [Bi Q, Wu Y, Mei S, Ye C, Zou X, Zhang Z, et al. Epidemiology and transmission of COVID-19 in 391 cases and 1286 of their close contacts in Shenzhen, China: a retrospective cohort study. The Lancet Infectious Diseases. 2020. pp. 911–919. doi:](http://paperpile.com/b/7MJin7/oTqR)[10.1016/s1473-3099(20)30287-5](http://dx.doi.org/10.1016/s1473-3099(20)30287-5)

4. [Lauer S, Truelove S, Kyra G. covidSeverity. 2020. Available:](http://paperpile.com/b/7MJin7/NsaR) <https://github.com/HopkinsIDD/covidSeverity>

5. [Lemaitre, J. C., Grantz K. H., Kaminsky J., Meredith H. R., Truelove S. A., Lauer S. A., Keegan L. T. et al. "A scena](https://doi.org/10.1038/s41598-021-86811-0)rio modeling pipeline for COVID-19 emergency planning." *Scientific reports* 11, no. 1 (2021): 1-13. doi:[10.1038/s41598-021-86811-0](https://doi.org/10.1038/s41598-021-86811-0)

6. Alharbi, N.K., Alghnam, S., Algaissi, A., Albalawi, H., Alenazi, M.W., Albargawi, A.M., Alharbi, A.G., Alhazmi, A., Al Qarni, A., Alfarhan, A. and Zowawi, H.M. “Nationwide seroprevalence of SARS-cov-2 in Saudi Arabia”. *Journal of infection and public health*, *14, no. 7* (2021): 832-838. doi:10.1016/j.jiph.2021.04.006
