# Supplementary material for: Effect of non-pharmaceutical interventions in the early phase of the COVID-19 epidemic in Saudi Arabia
Source: PLOS Glob Public Health. 2022 May 9;2(5):e0000237. doi: 10.1371/journal.pgph.0000237 (PMC10021433; doi:10.1371/journal.pgph.0000237)
Supplement: Attachment — Submitted filename: KSA_COVID_rebuttal.docx [file pgph.0000237.s008.docx]

**Reviewer #1**

This paper presents a mathematical modelling study about the spread of SARS-CoV-2 in Saudi Arabia during the first half year of the epidemic, focusing on the relative reduction in incidence during the different non-pharmaceutical interventions impelemented. The study uses a previously published mathematical model. The paper is well written and the methodology is appropriate.

We thank this reviewer for the positive evaluation of our work.

I have the following specific comments:
1) Although the model is already presented in a previous publication, it would be helpful to include a brief description also in this paper (which could be, for example, in the form of a graphical presentation showing the compartments). Although fine to keep the full parameterisation in the appendix, a few key values could also be presented in the main text, mentioning which parameters were fixed and which free.

We have expanded the methods section answering the specific points stated below:

l. 155: “*Fixed parameters include the infection fatality ratios, the incubation time, the serial interval, and the delays between infection and case confirmation and death, as detailed in Supplementary table Table S1.*”

Specific questions:
a) Were the compartments defined based on anything else than infection status and region (e.g., age?)

The compartments of the transmission dynamics model included susceptible, exposed, infected and recovered compartments and were not age-specific. We note however that modeled death estimates incorporate information on age-specific COVID-19 infection fatality rates and the age distribution of the population in each each region, as calculated by the [covidSeverity](https://github.com/HopkinsIDD/covidSeverity) R package (reference 22).

We have detailed this in the methods section:

l. 134: *“ The model consists of a compartmental SEIR model with three infected compartments representing transmission dynamics in each distinct region, with transmission occurring between locations as well. In this analysis we did not consider age stratification.”*

b) Were the first infections seeded into all regions, or did some regions receive their first cases within the model, by transmission within KSA?

Yes, all regions were seeded with cases.

We have added this detail to the methods:

l. 146: *“The number and timing of epidemic seeding events were estimated in each region by the model, with seeding being allowed in all regions. “*

2) Is it correct that you calibrated the model against confirmed cases and deaths (it is mentioned that these data were collected and the model was calibrated, but not explicitly using what indicators). Consider rephrasing.

Yes we fit the model to cases and deaths. We rephrased the methods to better reflect this:

l. 163: *“In this report we jointly calibrate the model to the observed weekly sums of confirmed cases and confirmed deaths at region-levels. We use a Poisson likelihood on the weekly number of reported cases and a square-root normal likelihood on the number of deaths as detailed in the Supplementary text S1.”*

Moreover, fitting a SEIR model against confirmed cases (that depend on a number of factors: true incidence, severity, testing policy, etc) is usually not a very robust choice. What was the assumption regarding the proportion of infections that are recorded as cases?

We agree that case data may have considerable noise and a reporting rate needs to be assumed. We assumed that each region had a different reporting rate, and inferred the reporting rates within the MCMC framework. We note that in the choice of likelihoods we give more weight to deaths than to cases to reflect our belief that death reporting has less bias. In particular, we used a Poisson likelihood on cases and a square-root normal likelihood on deaths:

$observedcases\sim Poisson\left( modeledcases \right)$

$\sqrt{\left( observeddeaths \right)}\sim Normal\left( \sqrt{\left( modeleddeaths \right)},\sqrt{\left( observeddeaths \right)}*0.075 \right).$

We have added these details to the supplementary material.

As noted in the discussion, a limitation of this approach is that we assume that reporting did not change in time. We stressed this limitation in the discussion.

l. 273: *“Finally, we did not incorporate changes to testing rates, death reporting rates, or the IFR of COVID-19 over time, which means that subsequent inference may not accurately represent transmission dynamics across different phases of the pandemic. ”*

3) As it seems, from end of May 2020 the restrictions were gradually relaxed. Did anything come in turn? For example, in most of Europe the systematic use of masks, regular testing, effective contact tracing, etc, started to be effectively used only after the initial lockdown.

Based on the information we received from World Bank collaborators, masking and testing policies did not change after the relaxation of the lockdown and curfews.

4) Second sentence of Results (Between 91%...) now slightly complicatedly formulated. What is the meaning? Do these refer to daily numbers of incident cases (e.g. in Al Jafw, 91% of the daily incident case numbers were within the modelled 95% CrI)? Referring to Comment 2, how is the simulated number of daily cases derived from the daily infections?

Yes, the interpretation of the sentence is correct. To clarify it we have rephrased it as follows:

l. 175: *“Modeled case and death trajectories matched observations in terms of the 95% credible intervals (CrIs), with 91% (Al Jawf) to 99.5% (Asir) coverage for incident cases and 92% (Al Madinah) to 100% (Al bahah, Al Hudud Ash Shamaliyah, Hail, Jizan, Najran) of incident deaths, ”*

As detailed above, modeled cases were based on model-estimated region-specific reporting rates, and related to observed cases assuming a Poisson distribution as detailed above.

5) The specificities of KSA as a setting could be discussed more. The differences observed between the regions could be worth discussion. Did the differences you observed in R0 reflect the expectations – for example, was the R0 higher in settings with large and densely populated cities?

We did expect R0 to vary between regions given the large difference in population density. We however did not find a systematic difference as shown below.

6) Figure 4 shows that – against what one would expect – Reff continued to decrease during the relaxation phases. During Phases I and II, this could be an artefact caused by the short duration of these phases; but in some regions, the Reff continued to decrease during Phase III. Do you have any potential explanations for this?

We agree that the continued decrease of Reff during Phase III is interesting. We do not have additional information that may explain this decline in Reff, but we surmise that fatiguing adherence of the population to NPIs or meteorological factors not captured by the NPI sequence may be driving these changes.

7) I could imagine that there is somewhat lower degree of social mixing in KSA that in many other settings, which limits the applicability of compartmental models that assume heterogeneous mixing within each region. This is a limitation that I think would be worth mentioning and discussing its implications.

We have added this limitation to the discussion.

l. 289: *“Moreover our model assumes homogeneous mixing of people with each region which may be a strong assumption in this specific analysis.”*

**Reviewer 2**

The manuscript presents a thorough analysis of NPIs in Saudi Ariabia, which adds to the growing literature in support of the impact of NPIs on transmission. While the approach is not novel, its application to a Middle Eastern country is, with the area as a whole significantly less studied than Europe or the Americas. This is a failure of the scientific community and as such I am very encouraged to see this analysis and thoroughly enjoyed reading it.

Overall, I am very happy with the analysis presented and the methods chosen. I have a few comments (somewhere between a minor comment and a major comment), which I think would enhance the manuscript and give more confidence in the findings presented:\

We thank this Reviewer for the positive evaluation of our work.

1. Seroprevalence data. A recent nationwide blood donor sample of seroprevalence was conducted in Saudi Arabia (Alharbi et al. 2021 July. [https://www.sciencedirect.com/](https://www.sciencedirect.com/science/article/pii/S1876034121000988)). While the bias in blood donor sample collection is an open question, it would be very useful to produce plots for each region of the seroprevalence over time inferred from your model fits and compare (if just qualitatively as opposed to formal model fitting) against the observed seroprevalence data. How well does it align and are some regions more aligned with seroprevalence than others?

We thank this Reviewer for pointing to the seroprevalence study. We show below the comparison between the seroprevalence estimates in the six sampled regions and their corresponding fraction of cumulative infections implied by our mode. The serosamples were collected from Jun-Nov 2020, depending on the region. Riyadh was sampled in Jun 2020. Modeled cumulative infection rates tended to underestimate serology-based estimates for both blood donor and overall samples, in particular for serosurveys done in the second half of the simulation period. We note that our simulation period ends in October 2020, whereas serosurveys in Ash Sharqiyah (Alahsa in Alhabri et al. 2021) and Makkah were done in November 2020 which may explain at least part of the underestimation. There is however a significant linear relationship between modeled infections and seroprevalence estimates, with approximately twice as many seroprevalence-based infections for each modeled infection. We believe that the systematic under-estimation could be caused by by an overestimation of the realized infection fatality ratio with respect to the estimates based on the region’s demographics, or the seroprevalence estimates could be over-estimated since the survey was based on a convenience sample.

**Figure 1: Comparison between seroprevalence studies and modeled infections.** Seroprevalence data was extracted from Alhabri et al. (2021), with estimates stratified by sampled population (blood donors and blood donors + non-COVId-19 patients). Cumulative modeled infections were matched to the midpoint of the serosurvey period when it fell within the simulation time window, and set to the end of the simulation period (Oct 31 2020) otherwise.

We have now mentioned this discrepancy in the discussion:

l. 267: *Seroprevalence estimates published after our modeling analysis suggest that the infection fatality ratios in regions of KSA may have been lower than the ones used in our analysis [38], although they were based on convenience samples not representative of the broader population.*

One reason for asking for this is because it would be useful to know if the regions where the model fit to cases incidence is weaker, e.g. Asir, are due to differences in case detection between region. For example, the ratio of deaths to case incidence seems quite consistent between regions but in some it is noticeably higher. Is this because of demographic factors or is it because of different cases detection and thus are the weaker case incident fits are to be expected but we can be confident in the fit to the deaths as the model fit inferred seroprevalence is in agreement with the observed seroprevalence. (Apologies for the longer comment, please feel free to break it down in reply).

The regions with poor concurrence between seroprevalence and modeled infection estimates, Al Quassim, Ash Sharqiyah, and Makkah, captured most of the observed case and death counts within the 95% credible interval, although the IQR projections were systematically lower than case observations during the summer peak. We recall that our IFRs are fixed according to demographic data and only case detection rates are allowed to vary by region and not in time. Thus, regional case detection rates would be sensitive to inflated or deflated IFR assumptions and may only capture case to infection ratios well during certain periods of the calibration. If regional population-representative seroprevalence estimates existed, we could use them to set better initial conditions for our inferred case detection rates, but the quality of our current fits suggests that the model infection dynamics are reasonably capable of describing the disease system. Ar

2. More information of the model likelihood and model fitting. Currently, it is hard to understand from the SI exactly what model outputs were used for fitting and what distributions were assumed for cases and deaths. E.g. were weekly sums of cases and deaths assumed to be described by a Poisson or Negative Binomial? The SI currency reads as a general overview of a model fitting framework, rather than specifically what the authors did in the analysis, e.g. in the following text:
“The framework allows the user to specify the time unit for a given calibration data; for instance one may calibrate to weekly incident deaths, biweekly confirmed cases, or both.”

We have expanded the methods as required by Reviewer 1 as well, as detailed above.

3. Please expand on this to describe exactly what model outputs were used to compare to the observed data and what distributions (your p_j terms) these were assumed to take.

We have detailed this in response to Reviewer 1 as detailed above.
In Table S1 please add the region specific estimates for each region as well as the region specific IFRs. Either as a sub table or a new table.

We have added a table with region-specific results in tables S3-S5.

4. In the fitting algorithm, the approach of multiple, shorter chains is fine, however, getting an unbiased estimator of the posterior this way is less valid if the initial conditions for each chain are the same. I assume they are not but it is not stated in the SI. Please add details of how each chain was initialised (the \Theta_prop terms)

Chains were indeed initialized independently between iterations following a random initial draw. We have added details in the supplementary material:

*Chains were initialized using random starting conditions for each parameter as follows:*

*R0 ~ truncated-normal(mean = 2, sd = .1, limits = [1, 3])*

*Isolate and test ~ truncated-normal(mean = .15, sd = .1, limits = [0, .75])*

*Curfew ~ truncated-normal(mean = .2, sd = .1, limits = [0, .9])*

*Lockdown ~ truncated-normal(mean = .7, sd = .1, limits = [0, .95])*

*Phase I ~ truncated-normal(mean = .3, sd = .1, limits = [0, .9])*

*Phase II ~ truncated-normal(mean = .15 sd = .1, limits = [0, .9])*

*Phase III ~ truncated-normal(mean = .6, sd = .1, limits = [0, .9])*

*case confirmation rate ~ truncated-normal(mean = .3, sd = .1, limits = [0, 1])*

5. Were any priors assumed for the NPI effect sizes as well as the seeding size and data? Were they all assumed to be flat and uninformative? If so please state this.

We used flat uniform priors on the NPI effect sizes and on the seeding dates, we set a normal prior on the basic reproductive number as detailed below, which we have added to the supplementary text:

*R0 ~ normal(2.5, 1)*

Other minor comments:

1. All the figures were very clear and it made reading the paper very enjoyable and easy to follow! (just wanted to pass that on)

We thank this Reviewer for their positive feedback, it is much appreciated.

2. For my own interest, I was wondering how much the between region migration fluxes changed model fitting and behaviour. If these were removed and each region was modelled independently, would different conclusions have been reached?

As mentioned in the discussion mobility fluxes in the model were based on April Facebook mobility data, which is a limitation of the study. In that period, mobility was much lower than pre-Covid-era estimates as seen in the Google mobility data for KSA. The weak mobility fluxes likely contributed an overall increase in transmission compared to an alternative model where each region was modeled separately. We do not expect the qualitative conclusions of the work to change had we assumed no mobility, although specific NPI effectiveness estimates may have slightly varied in both directions.
