# Supplementary material for: Effect of non-pharmaceutical interventions in the early phase of the COVID-19 epidemic in Saudi Arabia
Source: PLOS Glob Public Health. 2022 May 9;2(5):e0000237. doi: 10.1371/journal.pgph.0000237 (PMC10021433; doi:10.1371/journal.pgph.0000237)
Supplement: Attachment — Submitted filename: KSA_COVID_second_rebuttal.docx [file pgph.0000237.s009.docx]

We thank all reviewers for their contributions in improving the presentation of our work.

**Reviewer #2**

Related to the analysis of seroprevalence data. The figure showing the comparison of modelled infections is very interesting. The correlation is encouraging that the model is capturing the overall dynamics between regions well. This figure should be included in the SI. Also, when discussing the comparison against seroprevalence in the Discussion, the other explanation for the discrepancy is that deaths have occurred undetected and that the IFR is correct. This alternative explanation must also be listed next to the suggestion that the IFR is incorrect. I would argue strongly that deaths have been undercounted in Saudi Arabia (deaths were missed in all countries at the beginning of the pandemic) and that this contributes to the discrepancy as well, most likely more so than the IFR being overestimated in the current analysis

We thank this reviewer for the remark. We have now added the figure and a descriptive paragraph in the supplementary text, and have modified the discussion accordingly:

*l. 287-291: “Seroprevalence estimates published after our modeling analysis suggest that the infection fatality ratios in regions of KSA may have been lower than the ones we used in this analysis [38], or that there was a systematic under-reporting of deaths, although these seroprevalence estimates were based on convenience samples not representative of the broader population (S1 Text)”*
